# Marked central nervous system pathology in CD59 knockout rats following passive transfer of Neuromyelitis optica immunoglobulin G

**DOI:** 10.1186/s40478-017-0417-9

**Published:** 2017-02-17

**Authors:** Xiaoming Yao, Alan S. Verkman

**Affiliations:** Departments of Medicine and Physiology, University of California, San Francisco, CA USA

**Keywords:** NMO, Aquaporin-4, Complement inhibitor, Astrocyte, Complement-dependent cytotoxicity, Transgenic rat

## Abstract

Neuromyelitis optica spectrum disorders (herein called NMO) is an inflammatory demyelinating disease of the central nervous system in which pathogenesis involves complement-dependent cytotoxicity (CDC) produced by immunoglobulin G autoantibodies targeting aquaporin-4 (AQP4-IgG) on astrocytes. We reported evidence previously, using CD59^−/−^ mice, that the membrane-associated complement inhibitor CD59 modulates CDC in NMO (Zhang and Verkman, *J. Autoimmun.* 53:67–77, 2014). Motivated by the observation that rats, unlike mice, have human-like complement activity, here we generated CD59^−/−^ rats to investigate the role of CD59 in NMO and to create NMO pathology by passive transfer of AQP4-IgG under conditions in which minimal pathology is produced in normal rats. CD59^−/−^ rats generated by CRISPR/Cas9 technology showed no overt phenotype at baseline except for mild hemolysis. CDC assays in astrocyte cultures and cerebellar slices from CD59^−/−^ rats showed much greater sensitivity to AQP4-IgG and complement than those from CD59^+/+^ rats. Intracerebral administration of AQP4-IgG in CD59^−/−^ rats produced marked NMO pathology, with astrocytopathy, inflammation, deposition of activated complement, and demyelination, whereas identically treated CD59^+/+^ rats showed minimal pathology. A single, intracisternal injection of AQP4-IgG in CD59^−/−^ rats produced hindlimb paralysis by 3 days, with inflammation and deposition of activated complement in spinal cord, optic nerves and brain periventricular and surface matter, with most marked astrocyte injury in cervical spinal cord. These results implicate an important role of CD59 in modulating NMO pathology in rats and demonstrate amplification of AQP4-IgG-induced NMO disease with CD59 knockout.

## Introduction

Neuromyelitis optica spectrum disorders (herein called NMO) is an inflammatory demyelinating disease of the central nervous system with characteristic pathological features in spinal cord and optic nerves, and to a lesser extent in brain. Most NMO patients are seropositive for immunoglobulin G autoantibodies against astrocyte water channel aquaporin-4 (AQP4), called AQP4-IgG (or NMO-IgG) [13, 14]. The major pathological features in seropositive NMO include astrocyte damage, inflammation with prominent granulocyte and macrophage infiltration, vasculocentric deposition of activated complement, and demyelination, which can produce marked neurological deficits [10, 19, 26]. There is abundant evidence that pathogenesis in AQP4-IgG seropositive NMO patients involves AQP4-IgG binding to AQP4 on astrocytes and activation of the classical complement system, which causes complement-dependent cytotoxicity (CDC) leading to inflammation, blood–brain barrier disruption and demyelination [8, 13, 19]. Antibody-dependent cell-mediated cytotoxicity (ADCC) [24] and sensitized T cells [22, 35, 36] may also play a role in NMO pathogenesis.

Several lines of evidence implicate a major role for complement activation in NMO, including human pathology showing deposition of activated complement [16, 18, 26], rodent models showing complement-dependent NMO pathology following passive transfer of AQP4-IgG [1, 28, 37], and an open-label clinical trial of the C5 convertase inhibitor eculizumab showing efficacy in NMO [21]. We previously reported that complement inhibitor protein CD59, a phosphoinositol-linked membrane glycoprotein expressed on astrocytes that inhibits formation of the terminal membrane attack complex, may be an important regulator of complement action in NMO [38]. CD59^−/−^ mice are highly sensitive to administration of AQP4-IgG and human complement, with longitudinally extensive NMO spinal cord pathology produced by coinjection of AQP4-IgG and complement into the lumbosacral cerebrospinal space. However, a major limitation of mice as models of NMO is the near-zero activity of their classical complement pathway, in part because of complement inhibitory factor(s) present in mouse serum [25]. The ineffective classical complement pathway in mice precludes the development of clinically relevant NMO models, such as robust passive-transfer models of NMO optic neuritis and transverse myelitis, as well as testing of NMO therapeutics such as complement inhibitors.

To overcome these limitations and to further investigate the role of CD59 in NMO pathogenesis, here we generated CD59^−/−^ rats and determined their sensitivity to passive transfer of AQP-IgG. We previously showed that passive transfer of AQP4-IgG to rats, without added complement, by a single intracerebral injection produced NMO pathology in brain at the site of injection [1]. We tested here the prediction that marked NMO pathology might be produced in the central nervous system by passive transfer of AQP4-IgG to CD59^−/−^ rats, without added complement, under conditions where minimal pathology is produced in CD59^+/+^ rats.

## Materials and methods

### CD59^−/−^ rats

**Fig. 1:**
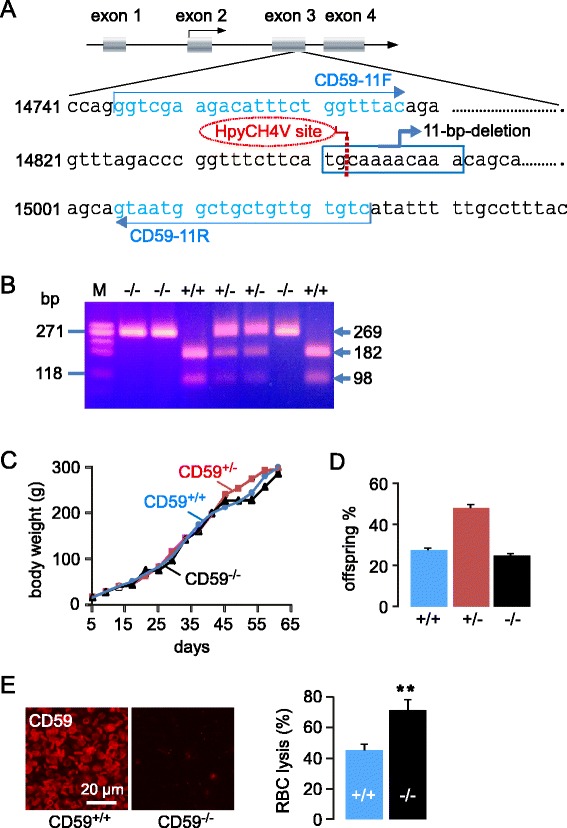
Generation and characterization of CD59^−/−^ rats. **a**. Schematic of CD59 gene deletion strategy showing deletion of 11 bp in exon 3, and PCR genotyping strategy. **b**. PCR genotype analysis following HpyCH4V restriction enzyme digestion, showing a single band at 269 bp for CD59^−/−^ rats, two bands at 182 bp and 98 bp for CD59^+/+^ rats, and three bands for CD59^+/−^ rats. **c**. Growth curves of CD59^+/+^, CD59^+/−^ and CD59^−/−^ rats (20 rats per group, differences not significant). **d**. Genotype distribution of 150 pups from breeding of CD59^+/−^ rats. **e**. (*left*) CD59 immunofluorescence in blood smears. (*right*) Percentage erythrocyte lysis following complement activation (mean ± S.E.M., *n =* 6, **P < 0.01)

CD59^−/−^ rats in a Sprague–Dawley background were custom-generated by Transposagen Biopharm. Inc. (Lexingtobon, KY) using CRISPR-Cas9 gene targeting technology. Exon 3 of the CD59 gene was targeted to induce sequence deletions with frame-shifts, which were identified by PCR genotyping and sequence analysis. Primers for PCR genotyping were: CD59-11 F (5′ to 3′: GGTCGA AGACATTTCTGGTTTAC) and CD59-11R (5′ to 3′: GACACAACAGCAGCCATTAC), followed by restriction enzyme digestion with HpyCH4V (Fig. 1a), which produced distinct bands corresponding to the wildtype and edited alleles (Fig. 1b). Breeding of CD59^+/−^ rats was done to generate wildtype (CD59^+/+^) and CD59^−/−^ rats for experiments. In vivo studies were done on 8- to 10-week-old, weight-matched CD59^+/+^ and CD59^−/−^ rats. Rats were maintained in air-filtered cages and fed normal rat chow in the University of California, San Francisco (UCSF) Animal Care facility. All procedures were approved by the UCSF Committee on Animal Research.

### Materials

Purified recombinant AQP4-IgG (rAb-53) was provided by Dr. Jeffrey Bennett (Univ. Colorado, Denver). Human complement was purchased from Innovative Research (Novi, MI) and human control IgG from Pierce Biotechnology (Rockford, IL). Unless otherwise specified chemicals were purchased from Sigma-Aldrich (St. Louis, MO).

### Astrocyte cell culture

Primary astrocyte cultures were generated from brain cortex of neonatal CD59^+/+^ and CD59^−/−^ rats at day 7 post birth, as described [15] with modification. Briefly, the cerebral hemispheres were isolated and cortical tissue was minced and incubated for 15 min at 37 °C in 0.25% trypsin-EDTA. Dissociated cells were centrifuged and resuspended in Dulbecco’s Modified Eagle’s Medium (DMEM) containing 10% FBS and 1% penicillin/streptomycin, and grown at 37 °C in a 5% CO_2_ incubator. After cell confluence (8–10 days), flasks were shaken in a rotator at 180 rpm overnight to purify astrocytes and medium was replaced with DMEM containing 3% FBS and 0.25 mM dibutyryl cAMP. Cultures were maintained for an additional 2 weeks. Cultures contained >95% astrocytes as shown by positive glial fibrillary acidic protein (GFAP) immunofluorescence.

### Complement-dependent cytotoxicity (CDC)

Astrocyte cultures were trypsinized and plated onto 96-well microplates at 20,000 cells/well and grown for 48 h. Human complement and AQP4-IgG were added in Hank’s balanced salt solution (HBSS, pH 7.2; Invitrogen), and cells were incubated at 28 °C for 2 h for cytotoxicity measurement by the Alamar Blue assay (Invitrogen) as described [38].

### Organotypic cerebellar slice cultures and ex vivo NMO model

Cerebellar slice cultures were prepared using an interface-culture method as described [34] with modification. Postnatal day 7 CD59^+/+^ and CD59^−/−^ rats were decapitated and the whole cerebellum was removed, placed in ice-cold HBSS and embedded in 2% low-melting point agarose. Parasagittal slices of 300-μm thickness were cut using a vibrating microtome and placed on transparent, non-coated membrane inserts (Millipore, Millicell-CM 0.4-μm pores, 30-mm diameter) in six-well plates containing 1 mL culture medium (50% MEM, 25% HBSS, 25% horse serum, 1% penicillin-streptomycin, 0.65% glucose and 25 mM HEPES), with a thin film of culture medium covering slices. Slices were cultured in 5% CO_2_ at 37 °C for 7 days with medium change every 2 days. AQP4-IgG (or control human IgG) and human complement were added on day 7 and slices were fixed 24 h later in 4% paraformaldehyde (PFA) for whole-mount immunostaining.

### Blood analysis

Blood (200 μL) was collected into EDTA tubes for cell analysis and into tubes without anticoagulant for serum. To study complement-mediated erythrocyte lysis, 100 μL of fresh rat serum was placed in wells of a 96-well plate and acidified by addition of 10 μL of 0.2 N HCl to each well to give a pH of 6.5-6.8, as described [23]. Erythrocytes (10 μL of 50% suspension in PBS) were added to each well, and hemolysis quantified by absorbance at 412 nm after 1 h incubation at 37 °C, referenced against zero and 100% lysis controls. Hematology parameters were measured using a Genesis Hematology Analyzer (Oxford Science, Oxford, CT).

### Intracerebral injection model

AQP4-IgG (30 μg) was delivered by intracerebral injection as described [1] with modification. CD59^+/+^ and CD59^−/−^ rats were anesthetized with ketamine (100 mg/kg) and xylazine (10 mg/kg) and mounted on a stereotaxic frame. A midline scalp incision was made and a burr hole of 1-mm diameter was drilled on each side of the skull 0.5 mm anterior and 3.5 mm lateral to the bregma. A glass pipette with 40-μm diameter tip was inserted at a depth of 5 mm to infuse AQP4-IgG (or control IgG) in a total volume of 3 μL over 10 min by pressure injection. After injection, the glass pipette was kept in place for 10 min before slow withdrawal (over 5 min) to prevent leaking. At day 7 rats were deeply anesthetized and transcardially perfused with 200 mL heparinized PBS and 200 mL of 4% PFA in PBS. Brains were removed and post-fixed for 4 h in 4% PFA and crytoprotected in 20% sucrose. Serial frozen coronal sections (thickness 7 μm) were cut on a cryostat.

### Intracisternal injection model

AQP4-IgG was delivered by injection into the cisterna magna of CD59^+/+^ and CD59^−/−^ rats. Rats were anesthetized as above, mounted on a stereotaxic frame, the cisterna magna was exposed, and a glass pipette with 40-μm tip diameter was inserted. AQP4-IgG or control IgG (15 or 30 μg in 10 μL artificial cerebrospinal fluid, aCSF) was infused at 2 μl/min over 5 min by pressure injection at 10 psi. In some experiments a recombinant monoclonal anti-AQP4 ‘aquaporumab’ lacking effector functions (AQP4-IgG^-CDC^) [30] was infused. After injection, the glass pipette was withdrawn with no leakage seen. At specified times, rats were euthanized as above, and brain, spinal cord and optic nerves were removed for sectioning. Rat motor function was scored as described [20] with modification: score 0 = normal movement; score 1, tail paralysis; score 2, hindlimb paralysis; score 3, hindlimb paralysis with frontlimb paresis; score 4, complete paralysis with moribund condition.

### Immunofluorescence

Cultured astrocytes and cerebellar slice cultures were fixed with 4% PFA for 15 min and incubated in blocking solution as described [34]. Frozen sections of brain, spinal cord and other organs were post-fixed with 4% PFA for 5 min and incubated in blocking solution as described [34]. Slides were then incubated for 2 h with antibodies against GFAP (1:200; Millipore), AQP4 (1:200, Santa Cruz Biotechnology), ionized calcium binding adaptor molecule 1 (Iba1; 1:400, Wako, Richmond, VA), CD45 (1:20, Cambridge, MA), C9neo (1:100, Santa Cruz Biotechnology), myelin basic protein (MBP, 1:100, Santa Cruz Biotechnology), human IgG (1:100, Santa Cruz Biotechnology), or CD59 (7A6, 5 μg/mL, LSBio, Seattle, WA), followed by the appropriate species-specific Alexa Fluor-conjugated secondary antibody for 1 h (5 μg/mL each, Invitrogen). Sections were mounted with VectaShield (Vector Laboratories, Burlingame, CA) for visualization of immunofluorescence on a Leica fluorescence microscope or Nikon confocal microscope.

### Statistics

Data are presented as mean ± S.E.M. Statistical analysis was performed using Prism 5 GraphPad Software package (San Diego, CA). The normality of the data was established by Bartlett’s test for equal variances and a one-way ANOVA with Newmann-Keuls post-hoc test to compare groups.

## Results

### Generation and characterization of CD59^−/−^ rats

CD59^−/−^ rats were generated as diagrammed in Fig. 1a. Following breeding and attempted expansion of 3 rat clones, we selected a clone that thrived well, which contained an 11-bp deletion in exon 3 of the rat CD59 gene. Genomic PCR analysis following restriction enzyme digestion of PCR products showed CD59^+/+^, CD59^+/−^ and CD59^−/−^ rats from breeding of CD59^+/−^ rats (Fig. 1b). CD59^−/−^ rats showed no overt phenotype, including neurological function and behavior, with similar growth found for CD59^−/−^ and CD59^+/+^ rats (Fig. 1c), and breeding of CD59^+/−^ rats gave an approximate 1:2:1 distribution of viable CD59^+/−^, CD59^+/−^ and CD59^−/−^ offspring (Fig. 1d). CD59 immunofluorescence in multiple tissues (blood smear shown in Fig. 1e, left) confirmed undetectable CD59 protein in the CD59^−/−^ rats. Functional studies of complement-mediated erythrocyte lysis confirmed the expected greater lysis in CD59^−/−^ compared to CD59^+/+^ erythrocytes (Fig. 1e, right). Hematological parameters suggested a mild hemolytic anemia in CD59^−/−^ rats as evidenced by reduced hematocrit and mild reticulocytosis (Table 1), as has been reported in humans lacking CD59 [12]. Peripheral tissues did not show inflammation, deposition of activated complement (C5b-9 immunofluorescence), or histological abnormalities (see below, and data not shown).

**Table 1:** Hematological parameters in CD59^−/−^ and CD59^+/+^ rats

	Hb (g/dl)	RBC (10^12^/L)	HCT (%)	RDW (%)	Retic (%)	WBC (10^9^/L)	Plt (10^9^/L)
CD59^+/+^	14.8 ± 0.5	8.7 ± 0.4	40.3 ± 1.5	13.3 ± 2.3	2 ± 0.1	7.5 ± 2.5	737 ± 165
CD59^−/−^	14.3 ± 0.2*	7.8 ± 0.2*	38.5 ± 1.2*	14.2 ± 2*	12 ± 0.1*	7.4 ± 3.1	729 ± 188

*Abbreviations*: *Hb* hemoglobin, *RBC* red blood cell count, *HCT* hematocrit, *RDW* RBC distribution width, *Retic* reticulocyte count, *WBC* white blood cell count, *Plts* platelet count

Mean ± S.D. of 6 rats per genotype (three males and three females)

*P <0.01 comparing CD59^−/−^ with CD59^+/+^

We note an interesting observation made in carrying out control studies (of AQP4-IgG administration to CD59^−/−^ rats) in which complement was inactivated by administration of cobra venom factor (350 units/kg), as we have done previously in CD59^+/+^ rats [1, 7]. All CD59^−/−^ rats receiving cobra venom factor became moribund and died within 12–24 h, whereas no abnormalities were seen in CD59^+/+^ rats treated identically.

**Fig. 2:**
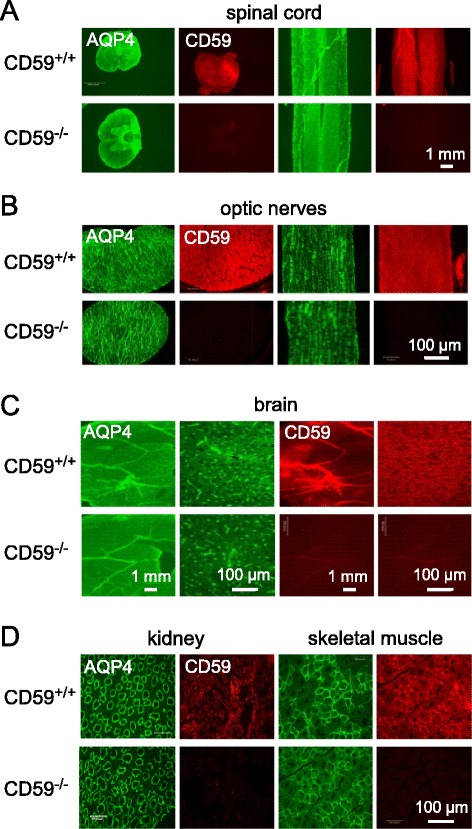
AQP4 and CD59 expression in CD59^+/+^ and CD59^−/−^ rats. Immunofluorescence shown in cross-section and longitudinal section of spinal cord (**a**), optic nerves (**b**), coronal sections of brain (**c**), and kidney inner medulla and skeletal muscle sarcolemma (**d**). Representative of two mice per genotype

Immunofluorescence of CD59 and AQP4 in CD59^+/+^ rats showed their gross coexpression in brain, spinal cord and optic nerve (Fig. 2a-c), in agreement with prior results [38]. We did not carry out high-resolution analysis of their cellular or subcellular localization. CD59 immunofluorescence of two major peripheral tissues in which AQP4 is expressed, kidney and skeletal muscle, also showed CD59 and AQP4 coexpression (Fig. 2d). CD59 immunofluorescence was not seen in CNS or peripheral tissues from CD59^−/−^ rats, and AQP4 immunofluorescence was similar in tissues from CD59^+/+^ and CD59^−/−^ rats.

### Marked complement-mediated injury in astrocyte cultures and brain slices from CD59^−/−^ rats

**Fig. 3:**
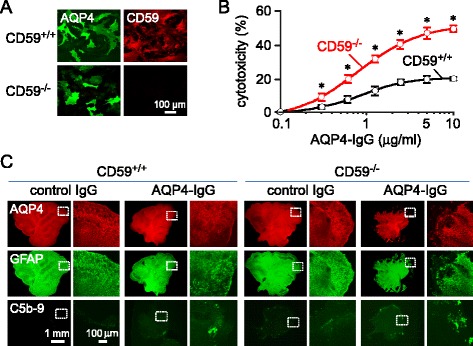
Complement-mediated injury in CD59^+/+^ and CD59^−/−^ astrocyte cultures and cerebellar slices. **a**. AQP4 and CD59 immunofluorescence in primary astrocyte cultures from neonatal CD59^+/+^ and CD59^−/−^ rats. **b**. Complement-dependent cytotoxicity in astrocyte cultures following 2-h incubation with 5% human complement and indicated concentrations of AQP4-IgG (mean ± S.E.M., *n =* 4, * *P <* 0.01). **c**. AQP4, GFAP and C5b-9 immunofluorescence in cerebellar slice cultures from CD59^+/+^ and CD59^−/−^ rats at 1 day after incubation with 5 μg/ml AQP4-IgG (or control-IgG) and 5% human complement. Fluorescence micrographs shown as low and high (boxed region) magnifications. Representative of 3 sets of slice culture studies

Complement-dependent cytotoxicity (CDC) was measured in primary astrocyte cultures generated from neonatal CD59^+/+^ and CD59^−/−^ rats. Immunofluorescence of astrocytes cultures from CD59^+/+^ rats showed CD59 coexpression with AQP4; similar AQP4 expression but without CD59 was seen on astrocytes from CD59^−/−^ rats (Fig. 3a). CDC was measured following 2-h incubation of astrocyte cultures with different concentrations of AQP4-IgG in the presence of human complement (Fig. 3b). CD59^−/−^ astrocyte cultures were significantly more sensitive to AQP4-IgG-induced CDC than were CD59^+/+^ astrocyte cultures, similar to prior results in CD59^+/+^ and CD59^−/−^ mouse astrocyte cultures [38].

To confirm the predicted greater sensitivity of a CD59^−/−^ CNS tissue to development of complement-mediated NMO-like pathology, ex vivo cultured cerebellar slices from CD59^+/+^ and CD59^−/−^ rats were incubated with AQP4-IgG and complement for 1 day. CD59^−/−^ cerebellar slices showed astrocyte injury with loss of AQP4 and GFAP immunofluorescence, seen most prominently at the peripheral border, and deposition of activated complement as seen by C5b-9 immunofluorescence (Fig. 3c). In contract, minimal loss of AQP4 and GFAP, and complement deposition were seen in CD59^+/+^ cerebellar slices under the same experimental conditions.

### Marked NMO pathology in brains of CD59^−/−^ rats following intracerebral AQP4-IgG injection

**Fig. 4:**
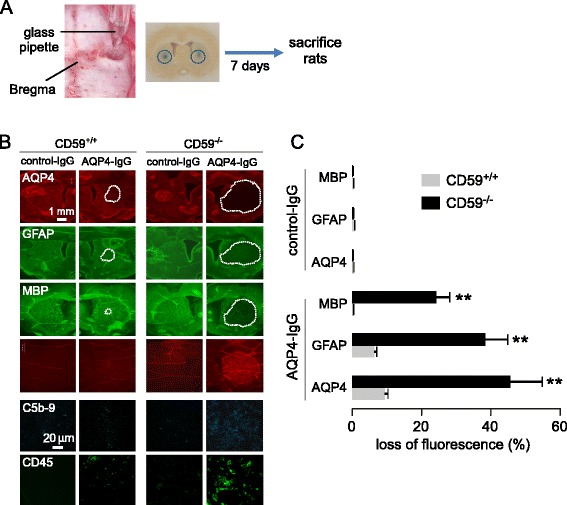
Increased NMO pathology in CD59^−/−^ rats following intracerebral injection of AQP4-IgG. **a**. Intracerebral injection model showing stereotaxic microneedle injection of AQP4-IgG (or control IgG). **b**. Immunofluorescence of indicated markers in rat brain at 7 days after AQP4-IgG (or control IgG) injection. Lesion areas indicated by white dotted boundary. **c**. AQP4, GFAP and MPB immunodeficient areas normalized to hemisphere areas (mean ± S.E.M., 6 rats per genotype, **P < 0.01)

Direct intracerebral injection of AQP4-IgG (without added complement) in rat brain has been shown to produce NMO-like pathology around the injection site, with loss of AQP4 and GFAP, deposition of activated complement, inflammation and demyelination [1]. To compare the sensitivity of CD59^+/+^ and CD59^−/−^ rats in this model, a submaximal amount of AQP4-IgG was injected into brain striatum (Fig. 4a). Under conditions in which minimal pathology was seen in CD59^+/+^ rats, there was marked astrocyte injury in the ipsilateral hemispheres of CD59^−/−^ rats as seen by loss of AQP4 and GFAP immunofluorescence and demyelination as seen by loss of MBP immunofluorescence (Fig. 4b), as well as microglia activation (Iba-1 immunofluorescence), leukocyte infiltration (CD45 immunofluorescence), and deposition of activated complement (C5b-9 immunofluorescence). Figure 4c summarizes areas of loss of AQP4, GFAP and MBP immunofluorescence in the ipsilateral, AQP4-IgG injected hemisphere and the contralateral, control IgG injected hemisphere. We conclude that CD59^−/−^ rat brain is highly susceptible to development of NMO pathology following exposure to AQP4-IgG.

### NMO pathology in CNS tissues of CD59^−/−^ rats following intracisternal AQP4-IgG injection

**Fig. 5:**
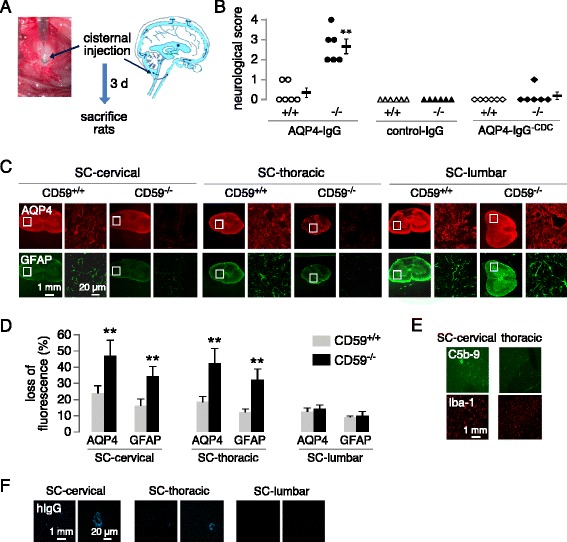
Increased NMO pathology in spinal cord of CD59^−/−^ rats following intracisternal injection of AQP4-IgG. **a**. Intracisternal model showing microneedle injection of AQP4-IgG (or control IgG). **b**. Neurological scores at day 3 after AQP4-IgG, control IgG, or engineered AQP4-IgG lacking complement effector function (AQP4-IgG^-CDC^). Each symbol is data from a separate rat (*n =* 6), with mean ± S.E.M. shown (**P < 0.01). **c**. Immunofluorescence of indicated markers in cervical, thoracic and lumbar spinal cord at 3 days after AQP4-IgG injection. **d**. Loss of AQP4 and GFAP immunofluorescence normalized to whole section area of spinal cord (mean ± S.E.M., 6 rats per genotype, **P < 0.01). **e**. C5b-9 and Iba-1 immunofluorescence in cervical and thoracic spinal cord at 3 days after AQP4-IgG injection. **f**. AQP4-IgG distribution at 2 h after intracisternal injection visualized with an anti-human secondary antibody

We next introduced AQP4-IgG into the CSF by intracisternal injection (Fig. 5a). In an initial study, injection of 30 μg AQP4-IgG into the CSF of CD59^−/−^ rats produced marked paralysis by day 1 and death soon thereafter. In subsequent studies, intracisternal injection of a reduced, 15 μg amount of AQP4-IgG produced motor dysfunction in all CD59^−/−^ rats by day 1, but with >80% survival on day 3; no hindlimb motor dysfunction or mortality was seen in CD59^+/+^ rats administered 15 μg AQP4-IgG or in CD59^+/+^ or CD59^−/−^ rats administered 15 μg control IgG or an engineered AQP4-IgG lacking complement effector function (Fig. 5b). Examination of skeletal muscle and kidney from AQP4-IgG treated CD59^−/−^ rats did not show C5b-9 deposition or inflammation, and human IgG was undetectable in the serum (data not shown), suggesting that the gross motor dysfunction in the antibody-treated CD59^−/−^ rats is not a consequence of peripheral organ injury.

Immunofluorescence of spinal cord on day 3 showed marked loss of AQP4 and GFAP in cervical spinal cord of AQP4-IgG-treated CD59^−/−^ rats, with patchy and variable loss in thoracic and lumbar spinal cord (Fig. 5c, d). Minimal loss of AQP4 and GFAP was seen in treated CD59^+/+^ rats. C5b-9 and Iba-1 immunofluorescence was seen in cervical and thoracic spinal cord in AQP4-IgG-treated CD59^−/−^ rats (Fig. 5e). To investigate whether the location-dependent pathology in spinal cord is related to AQP4-IgG access and deposition, tissues from rats receiving 15 μg AQP4-IgG were harvested at 2 h and immunostained with an anti-human secondary antibody. Figure 5f shows detectable human IgG in cervical > thoracic spinal cord, with little seen in lumbar spinal cord.

**Fig. 6:**
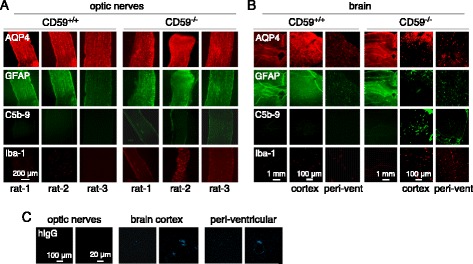
NMO pathology in optic nerves and brain of CD59^−/−^ rats following intracisternal injection of AQP4-IgG. **a**. Immunofluorescence of indicated markers in optic nerves at 3 days after AQP4-IgG injection. Data shown for 3 rats per genotype. **b**. Immunofluorescence of indicated markers near the brain surface (‘cortex’) and around ventricles (‘peri-vent’) at 3 days after AQP4-IgG injection. **c**. Distribution of AQP4-IgG at 2 h after intracisternal injection visualized with an anti-human secondary antibody

Immunofluorescence of optic nerves showed patchy and variable loss of AQP4 and GFAP (results from 3 rats per genotype shown in Fig. 6a); C5b-9 and Iba-1 immunofluorescence were consistently greater in the AQP4-IgG treated CD59^−/−^ rats than the CD59^+/+^ rats. Brain sections showed patchy loss of AQP4 and GFAP, mainly at the brain surface and in periventricular matter, along with C5b-9 and Iba-1 immunofluorescence (Fig. 6b). AQP4-IgG (human IgG immunofluorescence) at 2 h after injection was not detectable in optic nerves, but seen in a patchy distribution in brain cortex and periventricular matter (Fig. 6c).

## Discussion

Our study supports the central involvement of CD59 in modulating complement-mediated injury in AQP4-IgG seropositive NMO. CD59 is expressed in CNS tissues affected in NMO and may play a protective role to contain local, subclinical injury initiated by minor exposures to AQP4-IgG. CD59^−/−^ rats were highly sensitive to passive transfer of AQP4-IgG by intracerebral and intracisternal routes, without the need for added components such as complement or pro-inflammatory factors. Though astrocytes may also express other complement regulator proteins such as CD55, the marked effect of CD59 gene deletion suggests that CD59 is the major complement regulator in rat brain. As an important complement regulator in astrocytes, drugs that enhance astrocytic CD59 expression, perhaps identifiable by compound screens, may be beneficial in NMO, and conversely, reduced astrocytic CD59 expression or subcellular colocalization with AQP4 might trigger NMO exacerbations and worsen disease severity.

Animal models of NMO have been useful in characterizing NMO pathogenesis mechanisms and for testing potential NMO therapeutics. Mouse models have been developed involving passive transfer of AQP4-IgG together with human complement by direct injections into the brain [28] or spinal fluid [3, 35] to produce NMO-like pathology in brain, spinal cord and optic nerve. As mentioned in the Introduction, a fundamental limitation of mice to study NMO is their lack of an effective classical complement activation pathway [5, 25]. Early rat models involved administration of AQP4-IgG following induction of experimental autoimmune encephalomyelitis (EAE) [4]; however, the pathogenic mechanism in EAE – myelin targeting by T cells – is very different from the humoral immune response in NMO, making it difficult to reach conclusions about NMO pathogenesis mechanisms. We found that intracerebral injection of AQP4-IgG produced robust NMO-like pathology in rat brain [1], and that while systemic administration of AQP4-IgG alone did not produce disease, NMO-like brain pathology was seen following a small needle stab in seropositive rats [2], which presumably allowed circulating AQP4-IgG leakage into brain parenchyma to access astrocytes, and perhaps produce a local inflammatory response. Creation of NMO spinal cord or optic nerve pathology in rats has been challenging. One study involving continuous AQP4-IgG infusion using intrathecal catheters showed reversible AQP4 loss in spinal cord but without inflammation or demyelination [9], and a similar more recent study reported AQP4 loss in spinal cord and optic nerves, as well as mildly reduction in myelin in spinal cord [17]. The marked amplification of NMO pathology by knockout of CD59 in rats produced astrocytopathy as well as inflammation and deposition of activated complement.

CD59^−/−^ rats did not manifest overt phenotypes, except for mild reticulocytosis and reduced hemoglobin, which is likely due to low-grade hemolysis as seen in humans lacking CD59 [31] rather than a possible off-target effect in genome editing that can occur using CRISPR methods. The active classical complement system in rats, which has similar activity to that in human [5, 33], is presumably the reason for the low basal hemolytic activity. As such, CD59^−/−^ rats may be useful to model complement-initiated diseases in various neurodegenerative, hematological, renal and skeletal muscle diseases [6, 11, 31]. Although the mechanism of high morbidity in CD59^−/−^ rats receiving cobra venom factor was not established here, there appeared to be hemolysis and organ injury, which is likely due to complement activation and consumption by cobra venom factor, which is the mechanism of its complement depletion action [32, 33]. With regard to NMO, the amplified response of CD59^−/−^ rats to AQP4-IgG may be useful in testing drugs that target distinct steps in the AQP4-IgG/complement injury pathway, as well as in investigating outstanding questions in NMO pathogenesis mechanisms such as the role of sensitized T cells and the explanation for the absence of significant pathology in peripheral AQP4-expressing tissues despite their sustained direct exposure to serum AQP4-IgG.

The marked NMO pathology seen in CD59^−/−^ rats following AQP4-IgG administration contrasts with the conclusions of Saadoun and Papadopoulos [27], who concluded that complement inhibitors, including CD59, are not protective against complement injury in CNS tissues. Their findings were based on immunofluorescence in mouse brain in which CD59 expression was seen on astrocytes, but not at AQP4-rich foot-processes abutting microvessels. Detection sensitivity rather than species differences may account for the disparate conclusions, as we previously showed marked NMO pathology in CD59^−/−^ mice following intracerebral or lumbosacral administration of AQP4-IgG with human complement [38]. Our recent development of super-resolution microscopy methods to image AQP4 on astrocytes in fixed CNS tissues [29] may overcome the limited resolution and sensitivity of conventional fluorescence microscopy to detect CD59 in subcellular regions of astrocytes. Saadoun and Papadopoulos [27] also speculated that the absence of significant NMO disease in peripheral AQP4-expressing tissues such as skeletal muscle and kidney was a consequence of CD59 and AQP4 coexpression, which should be amenable to testing using CD59^−/−^ rats.

## Conclusion

In conclusion, our results implicate CD59 as an important regulator in NMO pathogenesis and potentially a new drug target with a novel mechanism of action to reduce complement-mediated astrocyte damage, a key initiating event in NMO. Prevention of complement-mediated astrocyte damage by altering astrocyte susceptibility to complement may have a more favorable side-effect profile than by general complement inhibition.
